# Visual Impairment Owing to Adverse Drug Reaction: Incidence and Routine Monitoring in the United Kingdom

**DOI:** 10.1016/j.ophtha.2013.12.022

**Published:** 2014-05

**Authors:** Phillippa M. Cumberland, Isabelle Russell-Eggitt, Jugnoo S. Rahi

**Affiliations:** 1 Medical Research Council (MRC) Centre of Epidemiology for Child Health at the Centre for Paediatric Epidemiology and Biostatistics, University College London (UCL) Institute of Child Health, London, UK; 2 Ulverscroft Vision Research Group, UK; 3 Great Ormond Street Hospital for Children NHS Foundation Trust, London, UK; 4 National Institute for Health Research (NIHR) Biomedical Research Centre at Moorfields Eye Hospital NHS Foundation Trust and UCL Institute of Ophthalmology, London, UK

Sight-threatening side effects of medication are rare, but can lead to a considerable individual and societal burden, especially when severe and/or permanent. Because these events are so uncommon, it is challenging to acquire data that clinicians can use to counsel patients or that identify novel potential risks. Currently in the United Kingdom, all serious suspected adverse drug reactions (ADRs) and any drug-related side effect of a new (black triangle) medication^1^ are “monitored” through the Medical and Health product Regulatory Agency (MHRA) using the voluntary Yellow Card Scheme^2^ to inform an anonymized national database. Classification of ocular ADRs is by eye condition with few categories indicating functional vision. Thus, it is not possible to estimate population incidence of visual impairment owing to ADRs through this source.

We therefore undertook a prospective observational study through the British Ophthalmological Surveillance Unit (BOSU), the long-established national scheme providing anonymized case ascertainment for epidemiologic studies of rare ophthalmic conditions of public health importance.^3^ We report on this first systematic national study of incidence of diagnosis of visual impairment owing to ADRs and comparative ascertainment by the MHRA scheme.

**Case Definition.** Any child or adult newly diagnosed with significant visual loss suspected to be owing to an ADR to any prescribed medication (topical or systemic),^4^ including, bilateral or unilateral visual impairment, severe visual impairment or blind, namely, distance acuity worse than Snellen 6/18 (logarithm of the minimum angle of resolution, 0.48) in the better eye if bilaterally affected or in the affected eye if unilateral (i.e., World Health Organization modified taxonomy), or eligible for certification as sight impaired (partial sight) or severely sight impaired (blind), based on visual fields. Patients with milder sight loss or those with raised intra-ocular pressure or cataract owing to steroid treatment (i.e., known and common dose-related side effects), were ineligible.

**Case Ascertainment.** Surveillance was undertaken over 24 months to February 2012, with 6-month follow-up data collection completed by November 2012. The BOSU sends a monthly report card to all consultant ophthalmologists in the UK listing all studies/conditions under surveillance. Ophthalmologists return the card either notifying a “case” or confirming they have no cases to report (active versus passive surveillance). The BOSU informs the study team, who send the reporting ophthalmologist a standardized data collection form. There is no direct patient contact. Completeness of ascertainment of eligible cases, evaluated in prior studies using multiple sources and capture–recapture analysis or other validation, ranges from 65% to 100% (personal communication, B. Foot, September 2013, Scientific Co-ordinator BOSU).^3^ There was no suitable additional source for the present study.

**Procedures.** At notification, information was requested on the patient’s visual status prior and post the suspected ADR, the specific ophthalmic condition attributable to the ADR, preexisting eye disease, all medications being used at the time, the name of the suspected drug, and its duration of administration, dose and administration route.

A follow-up questionnaire sent 6 months later collected information on both the permanency of the visual impairment reported and the probability of the causality of the ADR, using the World Health Organization-Uppsala Monitoring Centre (UMC) criteria. At follow-up, reporting clinicians were asked if the suspected ADR had also been reported additionally to the MHRA.

**Statistical Methods.** We report incidence based on cases with “permanent” visual impairment. For completeness, we report separately on “temporary” visual impairment, that is, patients whose visual function had improved above the study threshold at 6 month follow-up. Annual incidence rates were estimated using the Office of National Statistics 2011 Census population estimates for the UK as the denominator.

There were 36 eligible cases were reported between March 2010 and February 2012. Of these, 18 had permanent visual impairment confirmed (permanent cases), and 13 had temporary visual impairment. Outcome was not available in 5 cases, for various reasons.

The annual incidence of permanent visual impairment owing to suspected ADR was 0.2/1 000 000 adults (95% confidence interval, 0.10–0.37 per 1 000  000). Assuming the unconfirmed cases to be permanent, the estimate increases to 0.25 per 1 000  000 adults (95% confidence interval, 0.17–0.37), giving an estimated range for the annual incidence of 0.1 to 0.37 per 1 000 000 adults. There were no cases in children.

**Report to the MHRA (Yellow Card Monitoring Scheme).** Only 7 of the 17 permanent cases (41%) were definitely known to have been reported to the MHRA (1 case, no information). Five had not been reported and reporting status for 5 was unknown. Information was available for 9 of 13 of the temporary cases: 5 of the 9 (56%) were reported to the MHRA, 2 not reported, and 2 reporting status unknown. For drugs with well-established adverse ocular side effects, there was inconsistent reporting to the MHRA. For example, all permanent cases suspected to be owing to amiodarone (n = 2) or quinine (n = 2) were reported, but only 1 of 7 permanent cases suspected to be owing to ethambutol.

Our study serves to reassure clinicians and patients alike that vision impairment owing to ocular ADRs remains very uncommon, affecting <4 of every 10 million adults annually and is assumed to be even rarer in children. It also highlights the on-going challenges in monitoring ADRs that underlie the incomplete evidence base for recommendations about screening. Further research on individual drugs are necessary to address this.

Our findings suggest that currently the use of the MHRA Yellow Card Scheme for monitoring these events is inefficient. Notably, among the 236 reports of ADRs owing to ethambutol received by the MHRA since 1963, 4 (1.8%) reported optic neuropathy^5^; 3 of these were in the past 5 years, which suggests prior underreporting. Moreover, because 7 cases of optic neuropathy attributed to ethambutol were identified by our study over 24 months it is possible that active versus passive surveillance additionally prompted clinicians. If so, this suggests there is potential to increase the effectiveness of the MHRA Yellow Card Scheme by enhanced communication with clinicians.

For drugs that are known to have adverse ocular side effects or have known direct effects that can be reversed with immediate clinical management, reporting these events to the MHRA may not be the clinical priority. However, if the data collected were evaluated and reported back to clinicians regularly, there may be a better role for the MHRA system to monitor novel ocular ADR events, in partnership with clinicians and serve as a reliable sentinel surveillance system.
